# Clinical experience with multiplex ligation-dependent probe amplification for microdeletion syndromes in prenatal diagnosis: 7522 pregnant Korean women

**DOI:** 10.1186/s13039-019-0422-8

**Published:** 2019-02-26

**Authors:** Dongsook Lee, Sohyun Na, Surim Park, Sanghee Go, Jinyoung Ma, Soonha Yang, Kichul Kim, Seunggwan Lee, Doyeong Hwang

**Affiliations:** 1 Research Center of Fertility and Genetics, Hamchoon Women’s Clinic, 10, Seochojungang-ro 8-gil, Seocho-gu, Seoul, South Korea; 2 Department of Health and Environmental Science, Korea University, 145, Anam-ro, Seongbuk-gu, Seoul, South Korea

**Keywords:** Multiplex ligation-dependent probe amplification (MLPA), MLPA P245, Prenatal diagnosis, Increased nuchal translucency, Microdeletion syndromes

## Abstract

**Background:**

Conventional cytogenetic analysis using G-band karyotyping has been the method of choice for prenatal diagnosis, accurately detecting chromosomal abnormalities larger than 5 Mb. However, the method is inefficient for detecting the submicroscopic deletions and duplications that are associated with malformations and mental retardation. This study evaluated the results of the multiplex ligation-dependent probe amplification (MLPA) P245 assay used for prenatal diagnosis in cases with unusual ultrasonographic findings or specifically where parents wanted to be tested. The objective was to compare the results from MLPA with those from conventional cytogenetic testing in order to determine their concordance and the additional diagnostic yield of MLPA over G-band karyotyping.

**Results:**

Of the 7522 prenatal cases analyzed, 124 were found to have genomic imbalances (1.6%). Of those 124 cases, 41 had gene loss (33.6%), and 83 had gene gain (66.4%). Most of the cases with genomic imbalances (64.5%) showed no abnormal karyotype. In particular, all cases with a 4p16.3 deletion (Wolf-Hirschhorn syndrome) showed an abnormal karyotype, whereas all of those with a 22q11–13 deletion showed a normal karyotype. In most of the cases with pathogenic deletions, the indication for invasive prenatal testing was an increase in the nuchal translucency (NT) alone (51.2%). Other indications observed in the remaining cases were abnormal serum screening markers (14.6%), other ultrasonographic findings (9.8%), pregnancy through in vitro fertilization and fertility assistance (9.8%), and advanced maternal age(2.4%).

**Conclusions:**

These results show that for fetuses with an enlarged NT or abnormal ultrasonographic findings and normal conventional karyotype, additional genetic investigation like molecular testing would be for identifying the microscopic genomic aberrations (microdeletions, microduplications) responsible for syndromic associations including structural anomalies and mental retardation.

## Background

Cytogenetic analysis of fetal cells after chorionic villus sampling or amniocentesis is routinely offered to women who have an increased risk of carrying chromosomally abnormal fetuses. The indications for such prenatal diagnoses include advanced maternal age, increased risk for fetal trisomy identified by maternal serum screening, and fetal abnormalities detected through ultrasonography (USG). In the late first trimester of pregnancy, nuchal translucency (NT) is visible at the back of the neck in all fetuses, where an increased NT is often associated with chromosomal anomalies, especially Down syndrome [1, 2]. If the karyotype is normal but the NT is increased, the fetus has an increased risk of a wide range of congenital malformations, from isolated structural defects to genetic syndromes and neurodevelopmental delays to larger dilemmas of congenital anomalies [3–8].

The conventional cytogenetic test using G-band karyotyping has been the method of choice for prenatal diagnosis, accurately detecting chromosomal abnormalities larger than 5 Mb [9, 10]. However, it is inefficient for detecting the sub-microscopic deletions and duplications that are often associated with malformations and mental retardation. Multiplex ligation-dependent probe amplification (MLPA) can examine the subtle abnormalities that cannot be detected by conventional G-band karyotyping [11–14].

This study presents an overview of the results obtained from use of the MLPA P245 assay for prenatal diagnosis in cases with an unusual USG or in specific cases where parents wanted to be tested. The results from MLPA were compared with those from conventional cytogenetic testing in order to determine their concordance and the additional diagnostic yield of MLPA over G-band karyotyping.

## Results

Of the 7522 prenatal cases, 124 were found to have genomic imbalances (1.6%). Of those 124 cases, 41 were found to have gene loss (33.6%), whereas 83 cases had gene gain (66.4%) (Tables 1, 2, 3, and 4). Table 1 summarizes the indications for invasive prenatal diagnosis.

**Table 1:** Distribution of indications for genomic imbalances

Indications	Gene loss N (%)		Gene gain N (%)	
Increased NT	21	(51.2%)	34	(41%)
Serum screening(+)	6	(14.6%)	19	(22.9%)
Abnormal finding (USG)	4	(9.8%)	4	(4.8%)
Family history	2	(4.9%)	12	(14.5%)
Advanced maternal age	1	(2.4%)	7	(8.4%)
IVF & fertility assistance	4	(9.8%)	7	(8.4%)
Total	41		83	

[*NT* Nuchal Translucency, *IVF* in vitro fertilization, *USG* ultrasonography.] * When collecting indications, we were instructed to check only one item

**Table 2:** Detailed clinical data and karyotype results at the loss of genome

Deletion region				Karyotype result
1p36.33	1 (2.4%)			46,XY,der(1)t(1;9)(p36.3;q34.3)mat,
2q23.1	1 (2.4%)			46,XY
4p16.3 (Wolf-Hirschhorn syndrome)	8 (19.5%)			46,XX,der(4)t(4;18)(p15.3;q11.2)mat
				46,XX,der(4)t(4;6)(p12;q25)
				46,XX,der(4)t(4;18)(p15.3;q11.2)mat
				46,XX,del(4)(p14)
				46,XX,del(4)(p16.1)
				46,XX,add(4)(p15.2?)
				46,XY,der(4)t(4;21)(p16.1;q22.2)pat
				46,XX,del(4)(p15.2)
5p15(Cri du chat syndrome)	4 (9.8%)			46,XX,r(5)(?p15.5?q3
				46,XX,del(5)(p13)
				46,XX
				46,XY,der(5)t(5;6)(p13.3;p21.3)mat
5q35.3(Sotos syndrome)	3 (7.3%)			46,XX or 46,XY
15q11.2(Prader willi syndrome)	1 (2.4%)			mos 45,X [12]/46,X,Yq
16p13.3(Rubinstein-Taybi syndrome)	2 (4.9%)			46,XX or 46,XY
22q deletion	21 (51.2%)	22q11.21(DiGeorge syndrome)	16 (39%)	46,XX or 46,XY
		22q11.21 partial deletion	4 (9.8%)	46,XX,t(5;17)(q31.1;p13.3)
				46,XX or 46,XY
		22q13 partial deletion	1 (2.4%)	46,XY,t(17;18)(p13.3;q21.1)pat
Total	41

**Table 3:** Detailed clinical data and karyotype results at the gain of genome

Duplication region	N(%)			Karyotype results
1p36.33	3 (3.6%)			46,XX or 46,XY
3q29	13 (15.7%)			46,XX or 46,XY
				mos 45,X [14]/46,X,+mar [12]
				47,X,Yqh+,+ 21
				46,XX,der(10)t(3;10)(q21;p13)mat
				46,XY,add(8)(p21.3)
				mos 47,XY,15 ps+,+ 20 [14]/46,XY,15 ps + [36]
				46,XY,der(4)(pter→q?33::p?15.2 → pter)
				46,XX,der(21)t(4;21)(p16.1;q22.2)pat
				46,XX,+ 4,der(4)t(4;13)(q21.3;q12.3)pat.-13
5p15	2 (2.4%)			46,XY
				46,XY,t(4;5)(q25;p14.3)mat
7q11.23	3 (3.6%)			47,XX,+ 7
				46,XX
				mos 47,XY,+ 7 [39]/46,XY [11]
8q24	3 (3.6%)			47,XX,+der(14)t(8;14
				46,XY,der(20)t(8;20)(q23;p13)mat
				mos 47,XX,+ 8 [22]/46,XX [15]
9q22.3	2 (2.4%)			46,XY,der(9)inv.(9)(p12q13)t(9;13)(p22;q32)
				47,XX,+ 9
10p14	3 (3.6%)			46,XX,9qh+
				47,XX,+ 18
				mos 47,XY,+ 10 [43]/46,XY [3]
15q11.2	6 (7.2%)			
		15q11.2	4	46,XX or 46,XY
		15q24	2	46,XY
				46,XX,der(7)t(7;15)(q36.3;q15)
16p13.3	5 (6.0%)			mos 47,XY,+ 16 [24]/46,XY [7]
				46,XX or 46,XY
				47,XX,+ 16
17p13.3	4 (4.8%)			46,XX,t(2;8)(q35;q22)mat
				46,XX or 46,XY
17q21.31	1 (1.2%)			46,XY
22q	33 (39.8%)			
		22q11	15 (18.0%)	46,XX or 46.XY
				mos 47,XX,+ 7 [30]/46,XX [20]
				46,XY,1qh+,der(9)t(9;18)(p24;q21.3)pat
				47,XY,+der(22)t(13;22)(q34.1;q11.2)mat
		22q13	18 (21.7%)	46,XX or 46,XY
				47,XX,+der(22)t(20;22)(p13;q11.2)mat
				47,XY,+ 22
				47,XY,+ 18
				46,XY,der(15)t(15;22)(q26.1;q13.3)mat
				47,XX,+der(22)t(11;22)(q23.3;q11.2)mat
				mos 44,00,der(6)inv.(6)(p11.2q27)t(6;21)(q27;p11), \der(13;14)(q10;q10)pat [26]/45,00,der(13;14)pqt [16]
Xq28	1 (1.2%)			46,XX
Total	83

**Table 4:** Distribution of sample types used in this study

Tissue	Number of samples
Chorionic villus sampling*	4723
Amniotic fluid*	2796
Fetal blood*	22
Peripheral blood	36
Product of conception	30
Total	7607

[*prenatal samples: The mean gestational age at the time of test was 13.2 weeks for Chorionic villus sampling, 17.2 weeks for amniotic fluid, and 22.3 weeks for fetal blood

Most of the cases with genomic imbalances (64.5%) did not show an abnormal karyotype (Tables 2 and 3). In particular, all cases with a 4p16.3 deletion (Wolf-Hirschhorn syndrome) showed an abnormal karyotype, whereas those with 22q11–13 deletion showed a normal karyotype (Table 2).

In most of the cases with pathogenic deletions, the indication for invasive prenatal testing was an increased NT alone (51.2%); Other indications observed in the remaining cases were abnormal serum screening markers (14.6%), other findings from USG (9.8%), pregnancy through IVF and fertility assistance (9.8%), and advanced maternal age (2.4%) (Table 1). FISH results were available for 38 of 55 cases with pathogenic imbalances (data not shown).

## Discussion

Conventional cytogenetics has been the gold standard in prenatal diagnosis for detecting chromosomal abnormalities. At the light microscope level, the genome-wide numerical and structural anomalies can be examined and the resolution of chromosomal abnormalities of 5–10 Mb can be obtained [9, 10]. Indications for such prenatal diagnoses include advanced maternal age, increased risk for fetal trisomy identified by maternal serum screening, and fetal abnormalities (e.g., increased NT) detected through USG. There is much evidence that measurement of the NT alone or as part of a combined test is an excellent screening method for fetal aneuploidies, and an increased fetal NT with a normal karyotype is associated with an increased risk of adverse pregnancy outcomes [3–8].

Euploid fetuses with an increased NT may present with structural anomalies, including cardiac defects, diaphragmatic hernias, exomphalos, body stalk anomalies, and skeletal defects;, where certain genetic syndromes, (e.g., congenital adrenal hyperplasia, fetal akinesia, or Noonan syndrome), have been cited as possible causes. These present a huge dilemma in prenatal counseling. In particular, the 22q11.2 deletion syndrome (also known as DiGeorge or velocardiofacial syndrome) is the most common, with a reported prevalence ranging from 1 in 2000 – to 1 in 6000 live births. The frequency appears to exceed 1 in 1000 in referrals for prenatal diagnosis, even when cases with ultrasonographic evidence for fetal abnormalities are excluded. After Down syndrome, 22q11.2 deletion syndrome is the second most common cause of congenital heart disease and is an important consideration whenever a conotruncal cardiac anomaly is identified, in particular Tetralogy of Fallot. Additional developmental disabilities are often also present [15–17].

In this study, we present our experience of using the MLPA P245 assay during invasive prenatal diagnosis in 7522 selected cases with abnormal ultrasonographic findings or parents wanting to be tested. As a result, 124 cases were found to have genomic imbalances with 41 cases having gene loss (33.6%), and 83 having gene gain (66.4%) (Tables 2, and 3). The 22q11.2 deletion syndrome (39%) showed the highest frequency of 2.1 cases per 1000, and all had a normal karyotype (Tables 1, and 2). Conversely, all cases of 4p deletion (19.5%) were accompanied by chromosomal structural abnormality, and the same was true for the cases with a 5p deletion (9.8%) except for only one case. Indications were almost increased NT or ultrasonographic findings (Table 2). Most of the cases were found to have fetal malformations, but almost all of them had normal karyotypes. Thus, whereas deletions are clearly associated with clinical significance or genetic disease, in the cases of duplication, the clinical relevance still remains unclear (Tables 3), [18–22].

Five partial deletions of 22q (one deletion of the three probes) were detected (Tables 1, 2, and 3). MLPA analysis using parental DNA showed that one of the deletions was of maternal origin and two were of paternal origin, and they were expected to be consistent with the parent’s phenotype [23]. The other parents did not wish to tested. All cases were confirmed by the FISH method to be normal. FISH has been the gold-standard method for the diagnosis of microdeletion syndrome, but current FISH probes may not be able to detect all 22q11.2 deletions in the critical region; thus, it is possible that some cases that appeared to be false positive were in fact true positive [15–17].

As a consequence, in prenatal genetic counseling, clinicians faced with abnormal ultrasonographic finding, but normal karyotyping, find it difficult to predict the outcomes, and to offer proper genetic counseling and clinical management. Furthermore, the anxiety of prospective parents increases while they wait for the results of more highly informative testing [24–26]. In this study we used the MLPA P245 assay for our invasive prenatal chromosome test for a number of reasons. MLPA allows for a more comprehensive determination of gene dosages, with increased convenience and a relatively low cost compared with subtelomeric FISH analysis and other molecular methods. Moreover, MLPA can be used to detect micro-deletions that may not be identified by FISH, and it is more accurate for describing the karyotype owing to its delicate probe spacing. Therefore, the combined use of karyotype analysis and MLPA P245 in prenatal diagnosis facilitates greater accuracy and more informative results than those obtained from use of conventional cytogenetic methods only [27–34].

## Conclusions

In conclusion, for fetuses with an enlarged NT or abnormal USG and normal conventional karyotype, additional genetic investigations like molecular testing would be beneficial for identifying the microscopic genomic aberrations (microdeletions, microduplications) responsible for syndromic associations including structural anomalies and mental retardation.

## Methods

### Patients and samples

All samples from the 7522 pregnant Korean women enrolled in this study were received at the Research Center of Fertility & Genetics of Hamchoon Women’s Clinic between April 2010 and December 2017, for prenatal diagnosis using G-band karyotyping and the MLPA P245 assay. Details of the analyzed tissues are given in Table 4.

### Diagnostic techniques

Conventional chromosome analysis was performed on chorionic villi in accordance with the standard procedures used for evaluating numerical and structural chromosome aberrations in direct cytotrophoblastic cells preparations and long-term cultures of mesenchymal tissue (GTG-banding, 550 band level). Chromosome analysis using amniotic fluid and fetal blood was also performed according to standard protocols.

Genomic DNA for MLPA was extracted from T25-flask-cultured fetal cells using a QIAamp DNA kit (Qiagen, Hilden, Germany). The MLPA assay used to screen microdeletion syndromes was performed using the SALSA P245 probemix (MRC-Holland, Amsterdam, Netherlands) according to the manufacturer’s instructions. The amplification products were identified and quantified by capillary electrophoresis using a 3130 XL genetic analyzer (Applied Biosystems, Foster City, CA, USA). The data were analyzed with Gene Marker 1.85 software (SoftGenetics, State College, PA, USA).

Fluorescent in situ hybridization (FISH) was used to assess the structure of the disparity imbalance suspected of being pathogenic in the sample. FISH was performed on metaphase spreads on slides according to the manufacturer’s hybridization protocols.

## Abbreviations

FISH: Fluorescent in situ hybridization
MLPA: Multiplex ligation-dependent probe amplification
NT: Nuchal translucency
USG: Ultrasonography
